# Arrhythmias in Autoimmune Diseases: Immune-Mediated Mechanisms and Management

**DOI:** 10.3390/jcdd13070343

**Published:** 2026-07-22

**Authors:** Kamala P. Tamirisa, Jorge A. Irizarry-Caro, Ian Curnutt, Devika Adusumilli, Mia Jose, Meenakshi Jolly, Estelle Torbey, Annabelle S. Volgman

**Affiliations:** 1 Division of Cardiology, UT Southwestern Medical Center, Dallas, TX 75390, USA; 2 Division of Cardiology, UT San Antonio, San Antonio, TX 77030, USA; irizarrycaro@uthscsa.edu; 3 Department of Internal Medicine, Kansas City University College of Osteopathic Medicine, Joplin, MO 64804, USA; ian.curnutt@kansascity.edu; 4 Medical City Fort Worth, Fort Worth, TX 76104, USA; drdevika.adusumilli@gmail.com; 5 Senior High School, Carroll ISD, Southlake, TX 76092, USA; miajose28@gmail.com; 6 Division of Rheumatology, Rush University Medical Center, Chicago, IL 60612, USA; meenakshi_jolly@rush.edu; 7 Division of Cardiology, Brown University Medical Center, Providence, RI 02914, USA; estelle_t83@hotmail.com; 8 Division of Cardiology, Rush University Medical Center, Chicago, IL 60612, USA; annabelle_volgman@rush.edu

**Keywords:** rheumatoid arthritis and atrial fibrillation, systemic sclerosis and arrhythmias, arrhythmias in SLE

## Abstract

Autoimmune diseases substantially increase the risk of atrial and ventricular arrhythmias and sudden cardiac death through shared immune-mediated mechanisms. Pro-inflammatory cytokines, autoantibodies, and progressive myocardial fibrosis disrupt ion channel function, impair conduction, and create re-entrant substrates. Across systemic lupus erythematosus (SLE), rheumatoid arthritis (RA), and systemic sclerosis (SSc), these pathways manifest as atrial fibrillation, ventricular arrhythmias, conduction disease, and heightened arrhythmic mortality. Anti Ro/SSA antibodies, in particular, contribute to QT prolongation and atrioventricular (AV) block, while cytokines such as TNF α, IL 1β, and IL 6 remodel electrophysiological properties and promote fibrosis. Advanced cardiac imaging, especially cardiac magnetic resonance (CMR), detects inflammation and fibrosis even when ejection fraction is preserved, enabling earlier intervention. Management requires a dual approach: standard arrhythmia therapies alongside aggressive control of systemic inflammation with disease-specific immunosuppression. Despite growing evidence, major gaps remain, including the absence of randomized trials targeting immune-driven arrhythmias and the need for integrated risk models incorporating inflammatory biomarkers.

## 1. Introduction

Autoimmune and inflammatory diseases are increasingly recognized as contributors to cardiac arrhythmias and sudden cardiac death (SCD) [1]. Across autoimmune diseases, including systemic lupus erythematosus (SLE), rheumatoid arthritis (RA), and systemic sclerosis (SSc), immune-mediated injury creates the substrate for atrial fibrillation (AF), ventricular arrhythmias (VA), conduction system disease, and SCD [2,3]. For instance, in SLE, the 10-year risk of VA, SCD, or implantable cardioverter defibrillator (ICD) implantation is nearly three times that of the general population [4]; similarly, patients with RA are at twice the risk for SCD compared to those without the disease [3].

This review focuses on immune-mediated mechanisms of arrhythmogenesis in SLE, RA, and SSc as prototypical autoimmune rheumatic diseases with distinct pathophysiologic arrhythmogenic mechanisms, and their clinical management. Cardiac sarcoidosis, which has a well-established granulomatous arrhythmogenic substrate and is addressed by recent state-of-the-art reviews and statements, warrants a separate dedicated review [5].

## 2. Immune-Mediated Arrhythmogenesis

Immune-mediated arrhythmogenesis in autoimmune diseases can be grouped into three overlapping mechanisms. First, pro-inflammatory cytokines, particularly tumor necrosis factor-α (TNF-α), interleukin-1β (IL-1β), interleukin-6 (IL-6), and interleukin-17 (IL-17), disrupt cardiac ion channel function, prolonging action potential duration and promoting triggered activity and re-entry [2,6,7]. The sustained release of IL-1β and IL-18 drives immune cell recruitment and fibroblast activation via nod-like receptor protein 3 (NLRP3) inflammasome activation [8,9]. Second, circulating autoantibodies exert direct electrophysiologic effects on the myocardium; anti-Ro/SSA antibodies inhibit hERG potassium channels, contributing to QT prolongation and increased vulnerability to VA, while gain-of-function mutations in CACNA1C (L-type calcium channel) lead to QT prolongation and increased vulnerability to VA [8,10]. A population-based study of more than 17,000 anti-Ro seropositive individuals found significantly higher rates of both conduction disturbances (odds ratio (OR) of 1.44; 95% confidence interval (CI) of 1.25–1.66) and rhythm disturbances (OR of 1.21; 95% CI of 1.11–1.31) [9]. Third, inflammation drives fibrosis, mediated by transforming growth factor-beta 1 (TGF-β1) and nuclear factor kappa-light-chain-enhancer of activated B cells (NF-κB), and produces zones of heterogeneous conduction, providing substrate for re-entrant circuits [2,7,8]. 

## 3. Immune Signaling as an Arrhythmogenic Substrate

The immune system modulates cardiac electrophysiology (EP) at multiple levels, from ion channel functions to intercellular coupling. Understanding these mechanisms is essential because they operate across all inflammatory and autoimmune cardiac diseases, creating a shared arrhythmogenic pathway.

### 3.1. Cytokine-Mediated EP Remodeling

Pro-inflammatory cytokines directly alter cardiomyocytes’ EP properties [9]. TNF-α downregulates Kv4.2/4.3 (reducing Ito) and inhibits both IKr and IKs, collectively prolonging action potential duration [11]. IL-1β increases spontaneous sarcoplasmic reticulum calcium release, facilitating early and delayed afterdepolarizations while IL-6 enhances L-type calcium current (ICaL) via Cav1.2 phosphorylation and impairs sarcoplasmic reticulum Ca^2+^-ATPase 2a (SERCA2a)-mediated calcium reuptake [7]. Computational models confirm that these cytokines alter repolarization heterogeneity and lead to prolonged QTc on the surface ECG [12]. Furthermore, NLRP3-mediated IL-1β secretion contributes to VA through potassium current reduction and CaMKII oxidation, forming a “Coumel’s triangle” where inflammation is the trigger, fibrosis is the substrate, and autonomic imbalance is the modulator [13].

### 3.2. Structural and Gap Junction Remodeling

TGF-β1 is the principal pro-fibrotic mediator, stimulating fibroblast-to-myofibroblast differentiation, and TNF-α amplifies this process by activating TGF-β/Smad2/3 signaling, creating a feed-forward inflammatory–fibrotic loop [7,14]. TGF-β1 increases gap junctional coupling between myofibroblasts and cardiomyocytes more than five-fold via Cx43 upregulation, resulting in fibrotic changes and heterogenous impulse propagation [15,16]. These properties predispose tissue to re-entry in autoimmune diseases. Recently, cardiac resident macrophages were framed as essential participants in maintaining conduction by promoting Cx43 phosphorylation, and loss of this pathway causes VA and SCD in autoimmune diseases [17].

## 4. Autoimmune Diseases, Pericardial Syndromes, and Arrhythmias

Autoimmune diseases are linked to a 1.4-to-3.6-fold increase in cardiovascular risk, including arrhythmias, compared with the general population. This excess risk is particularly pronounced in adults younger than 45 years (hazard ratio (HR) of 2.33; 95% CI of 2.16–2.51) [18]. AF is the most common arrhythmia across most autoimmune conditions, except SSc, where VA is more prevalent [2]. Similarly, among patients with pericardial syndromes, the risk of arrhythmias is non-negligible. In patients with acute pericarditis, the rate of AF or atrial flutter was reported to be ~8–10% [19,20,21], though higher rates have been reported. Ventricular arrhythmias, on the other hand, are more common in patients with myocardial inflammation complicating acute pericarditis (myopericarditis) [22]. 

### 4.1. Systemic Lupus Erythematosus

SLE is associated with a broad spectrum of arrhythmias, including sinus tachycardia, AF, VA, and conduction system disease [23]. In a Danish cohort of over 3000 SLE patients, the 15-year absolute risk of the composite of VA, SCD, and ICD implantation was nearly three-fold higher than in the general population, as was the absolute risk of AF/AFL [4]. The arrhythmogenic substrate in SLE is multifactorial myocarditis creating ischemic and non-ischemic scars for VA; small vessel vasculitis and infiltration of the conduction tissue can cause varying degrees of atrioventricular (AV) block in 34–70% of patients [24]. Unlike RA, conduction abnormalities in SLE may regress when the underlying disease is controlled [24]. Anti-Ro/SSA antibody positivity in SLE is an independent risk factor for QTc prolongation, congenital AV block and VA [25,26]. These antibodies inhibit the hERG potassium channel through cross-reaction with its extracellular pore region, prolonging the action potential duration (APD) and causing acquired long-QT syndrome [24]. Anti-Ro/SSA antibodies also block L-type calcium channels in the AV node, representing a potentially reversible cause of heart block in adults [27]. In a large population-based study of over 17,000 anti-Ro seropositive subjects, seropositivity was associated with significantly higher rates of conduction disturbances (OR of 1.44; 95% CI of 1.25–1.66), independent of concurrent autoimmune disease [27]. Among US veterans, anti-Ro/SSA positivity was independently associated with QTc prolongation (OR of 2.27 for QTc > 500 ms; 95% CI of 1.34–3.87) after adjustment for QT-prolonging drugs [28]. These findings suggest that anti-Ro/SSA antibodies may silently contribute to VA and SCD even in the general population [29]. Hydroxychloroquine, a cornerstone of SLE therapy, has been associated with QTc prolongation, particularly in the presence of other QT-prolonging medications or underlying cardiac risk factors [27]. Hydroxychloroquine may also diminish the risk of AF in these patients [30].

### 4.2. Rheumatoid Arthritis

RA patients face twice the risk of SCD compared with non-RA subjects, pointing to an increased propensity for malignant VA and QT prolongation [3]. Large population-based studies, such as the Danish nationwide cohort of 18,247 RA patients showed an adjusted incidence rate ratio of 1.41 (95% CI of 1.31–1.51) for AF compared with the general population [31]. Similarly, the Korean nationwide study of 48,885 RA patients found an adjusted HR (aHR) of 1.55 (95% CI of 1.46–1.65) for AF with seropositive RA carrying a higher risk than the seronegative disease (aHR of 1.63 vs. 1.37) [29]. The underlying mechanisms are likely both indirect, through premature ischemic heart disease, small vessel disease, and heart failure, and direct, through cytokine-mediated effects on cardiac electrophysiology [27]. It is shown that tight control of disease activity may represent in early stages the most effective intervention to reduce arrhythmic risk in RA [31].

### 4.3. Systemic Sclerosis

SSc carries the highest cardiovascular risk among autoimmune diseases (HR 3.59; 95% CI 2.81–4.59), and cardiac involvement accounts for a significant proportion of SSc-related deaths, with ominous prognosis [25,32]. Microvascular coronary disease causes ischemia-reperfusion injury, leading to myocardial inflammation, cardiomyocyte apoptosis, and subsequent fibrosis [33]. Early SSc arrhythmogenesis appears to be driven predominantly by inflammation, particularly myocarditis. Supporting this concept, Bairkdar et al. demonstrated that arrhythmia incidence was highest within the first year following SSc diagnosis, with an approximately 2-fold increased risk compared with matched controls [34]. Over time, SSc progresses from an inflammatory phenotype toward focal myocardial injury and replacement fibrosis. Focal myocardial lesions, ranging from contraction band necrosis to replacement fibrosis without coronary artery abnormalities, are seen in approximately 50% of SSc patients, attributed to vasospasm (beyond small vessel disease involving digits) [34,35]. This patchy fibrosis serves as the primary substrate for re-entrant circuits. Premature ventricular contractions are the most frequent rhythm disturbance, reported in up to 67% of patients, while non-sustained ventricular tachycardia occurs in 7–13% and SCD in 5–21% of unselected patients [36]. AF, atrial flutter, or paroxysmal supraventricular tachycardia is also described in 20–30% of SSc patients [33]. Conduction disturbances due to sinoatrial node fibrosis, including bundle and fascicular blocks, occur in 25–75% of patients [33]. Cardiac magnetic resonance (CMR) with non-junctional fibrosis is seen in approximately 25% of SSc patients presenting with VA, and a history of digital ulcers and VA were independent predictors of myocardial replacement fibrosis [37]. Treatment could potentially decrease the risk of arrhythmia development, but care should be taken in monitoring the side effects of arrhythmias caused by medical therapy, notably cyclophosphamide. Thus, mycophenolate mofetil is more favored as first-line therapy due to its cardiac safety profile and efficiency [38]. 

### 4.4. Pediatric Considerations: Neonatal Lupus and Congenital Heart Block

Maternal anti-Ro/SSA (and to a lesser extent anti-La/SSB) antibodies cross the placenta and cause autoimmune-medicated CHB in approximately 2% of anti-Ro/SSA-positive pregnancies [39,40]. The anti-Ro/SSA antibodies block L-type calcium channels in the fetal AV node, causing progressive conduction injury [27]. Once CHB develops, spontaneous reversal is rare, and many affected infants require permanent pacemaker implantation [40].

Regarding steroid therapy, the American College of Rheumatology conditionally recommends oral dexamethasone 4 mg daily for fetal first- or second-degree heart block detected on echocardiography but conditionally recommends against dexamethasone for established complete heart block without other cardiac inflammation [41]. The PRIDE study and subsequent registry data have shown inconclusive results for steroids in preventing progression to complete block [39]. The PATCH study, a prospective open-label trial, demonstrated that HCFIGUREQ 400 mg daily initiated by 10 weeks of gestation reduced CHB recurrence from an expected 18% to 7.4% in anti-SSA/Ro-positive mothers with a prior affected child [42]. More recently, rozanolixizumab, a humanized anti-FcRn monoclonal antibody, was used in a proof-of-concept case to reduce maternal anti-Ro/SSA antibody transfer by ~65%, resulting in normal fetal cardiac conduction despite three prior neonatal lupus-affected pregnancies [43].

In the fetus, these antibodies cause irreversible conduction system damage (fibrosis of the AV node), whereas in adults, anti-Ro/SSA-associated AV block may be potentially reversible with immunosuppressive therapy, as the mechanism involves functional calcium channel blockade rather than permanent structural injury [27].

### 4.5. Juvenile Rheumatic Disease

For juvenile rheumatic diseases more broadly, pericarditis is the most common cardiac manifestation in systemic juvenile RA and pediatric lupus, while arrhythmias are less frequent but do occur [44]. Conduction defects are particularly notable in juvenile mixed connective tissue disease and juvenile systemic sclerosis [44]. Systemic juvenile RA can involve all cardiac structures including the conduction system, and subclinical cardiac involvement may be underrecognized [45].

### 4.6. Other Autoimmune Diseases

Beyond SLE, RA, and SSc, myocarditis-associated arrhythmias are well recognized in other autoimmune diseases. In idiopathic inflammatory myopathies (IIMs), cardiovascular disease has emerged as a leading cause of mortality, with myocarditis present in up to 38% of patients at post-mortem and severe arrhythmias reported in over 50% of those with myocardial involvement [46]. AF, QTc prolongation, and conduction abnormalities are more prevalent in IIM patients than healthy controls [47]. Among the vasculitides, eosinophilic granulomatosis with polyangiitis (EGPA) carries the highest risk of cardiac involvement, with cardiomyopathy in up to 40% of patients, and complete heart block was reported [48]. A detailed discussion of these conditions is beyond the scope of this review.

### 4.7. Autoimmune Pericardial Syndromes

Pericarditis is a common cardiac manifestation of SLE, RA, and SSc, and may be complicated by arrhythmias. The link between inflammatory acute pericarditis and arrhythmias was first described in 1956 where AF was reported in 13% of patients with acute pericarditis [49]. The mechanism by which arrhythmias develop in pericarditis remains controversial [22]. Some studies suggest that inflammation in pericarditis propagates to the conduction system, leading to conduction disorders (varying degrees of AV block and complete heart block) [22,50]. Another study found sinus node involvement in all cases (38 out of 38) of patients with acute inflammatory pericarditis who died of conduction disturbances, including complete heart block [22,51]. In patients with autoimmune pericarditis, conduction disorders were associated with atrial arrhythmias in approximately 8–10% of patients [19,20,21]. Risk factors associated with the development of atrial arrhythmias in pericarditis, applicable to autoimmune etiologies, include older age [20,52] and presence of hypertension, pericardial effusion and dilated left atrium [22,52]. Patients with pre-existing AF or atrial flutter had a higher rate of AF recurrence [52]. 

Myopericarditis is characterized by the inflammation of the pericardium with myocardial involvement. Arrhythmogenesis in myopericarditis is multifactorial and may result from mechanisms including ischemia secondary to coronary macrovascular dysfunction, impaired electrical conduction due to gap junction dysfunction, and T-cell-mediated autoimmunity against the myocardium [53]. Clinically, myopericarditis primarily presents with pericardial symptoms accompanied by troponin elevation and has been observed in approximately 14–32% of acute pericarditis cases [22,54,55]. One study reported that patients with myopericarditis (14.6%, 40/274) had a significantly higher frequency of cardiac arrhythmias compared with patients with acute pericarditis (65% vs. 16.7%, *p* < 0.001) [53]. Paroxysmal supraventricular tachycardia (excluding AF/flutter) and ventricular arrhythmias have also been more frequently associated with myopericarditis compared to acute pericarditis [50,55].

Cases of AF/flutter have also been described in the literature among patients with cardiac tamponade, a life-threatening complication of pericarditis that can lead to cardiogenic shock and increased mortality [22,56,57,58]. In some of these cases, the atrial arrhythmia can be resolved once pericardiocentesis is performed [56]. 

Atrial arrhythmias, most commonly AF, is the most common arrhythmia in patients with constrictive pericarditis, which complicates <1% of idiopathic/viral pericarditis and approximately 8% of specific pericarditis cases [22,59]. Recurrent pericarditis, which is more common in autoimmune-associated cases, may further increase the cumulative risk of atrial arrhythmias and constrictive physiology. A previous study found 22% of patients (two out of nine) with constrictive pericarditis developed atrial arrhythmias [59]. The prevalence of these arrhythmias depends mainly on the disease stage [22]. Persistently elevated right atrial pressure may account for atrial arrhythmias in advanced stages of the disease [22,60]. While most data on pericarditis-associated arrhythmias derive from mixed or idiopathic cohorts, the mechanisms and risk factors described above are likely applicable to autoimmune-mediated pericarditis, though dedicated studies in this population remain limited.

## 5. Diagnostic Tools

Multimodality imaging enables detection of the inflammatory and fibrotic substrates in autoimmune diseases, even before clinical events occur. Thus, the European Association of Cardiovascular Imaging (EACVI) consensus document emphasizes the role of early imaging and intervention in patients with autoimmune diseases [61]. 

### 5.1. Electrocardiogram (ECG) and Holter Monitors

ECG is an inexpensive and noninvasive test that can detect cardiac abnormalities with important prognostic implications [62]. Conduction disturbances, such as bundle branch block and AV block, are common in patients with autoimmune diseases with a prevalence of 5 to 35% [63,64]. A pooled analysis from a U.S. Preventive Services Task Force (USPSTF) systematic review on screening asymptomatic adults with a resting ECG found that abnormalities such as ST-segment or T-wave abnormalities, left ventricular hypertrophy, bundle branch block, or left axis deviation were associated with an increased risk for subsequent cardiovascular events after adjusting for traditional risk factors [65]. Among patients with SLE, a study found that the most common ECG abnormalities observed were abnormal ventricular repolarization (39.5%), which mostly consisted of T-wave changes, nonspecific ST-segment changes, and prolonged QT interval [62]. Another study found that the prevalence of an abnormal baseline ECG was greater in SLE patients compared to the general population (prevalence of 3.6–17%) [66]. Furthermore, abnormalities in the baseline ECG were associated with lupus disease activity and damage [66]. On the other hand, routine ECGs may not be a helpful screening tool in patients with asymptomatic SSc. A study found no difference in arrhythmia (NSVT or PVCs) or conduction abnormality between patients with SSc and controls (with and without stratification by age) [67]. Nevertheless, ECGs remain an important tool in the assessment of arrhythmias and conduction abnormalities in patients with autoimmune disease who are particularly susceptible to autoantibody damage of their conduction system.

Holter monitors are additional diagnostic tools that can be considered in the identification of conduction abnormalities and arrhythmias among patients with connective tissue diseases. A previous study comparing 24 h Holter monitoring in patients with SSc and SLE found that patients with SSc more frequently presented with atrioventricular and intraventricular conduction disturbances as well as non-sustained ventricular tachycardias when compared to patients with SLE [68]. By contrast, in SLE, QTc interval prolongation and short supraventricular tachycardias were more frequent [68]. 

### 5.2. Echocardiogram

Conventional echocardiography underestimates subclinical myocardial involvement in autoimmune diseases, because left ventricular ejection fraction (LVEF) may remain preserved despite significant disease [36]. However, speckle-tracking echocardiography helps overcome this limitation by quantifying myocardial dysfunction, such as reduced global longitudinal strain (GLS), and right ventricular and radial strain [36,69]. Left ventricular GLS correlates with SLE disease severity, and independently predicts cardiovascular events, despite normal LVEF [70]. Speckle-tracking echocardiography appears to be a first-line screening tool for early myocardial disease and arrhythmia risk prediction across all autoimmune diseases [71]. 

### 5.3. Cardiac MRI (CMR)

CMR provides tissue characterization, including edema (T2-weighted), diffuse fibrosis (T1 mapping), and replacement fibrosis (delayed enhancement), without needing ionizing radiation. In SLE, native T1 and T2 values are elevated compared with controls, with elevated T2 signals correlating with disease severity, and delayed enhancement is seen in 38 to 69% of patients. In RA, native T1 correlates with disease activity linking systemic inflammation directly to myocardial disease [72]. In SSc, delayed enhancement is present in around 18 to 53% of patients, directly correlating with VA [73]. Delayed enhancement patterns follow mostly non-ischemic patterns, with subepicardial and mid-myocardial uptake reflecting inflammatory etiology, rather than ischemic injury [61]. Even when LVEF is normal, delayed enhancement and abnormal T1 and T2 signals are significantly associated with arrhythmias and remain independent predictors for poor outcomes in this population [36]. Different patterns of LGE have been described but none are specific to the disease. In conclusion, CMR remains an important imaging modality to guide early intervention before irreversible fibrosis and arrhythmias develop in these patients.

### 5.4. Endomyocardial Biopsy

Endomyocardial biopsy (EMB) remains the gold standard for histopathological diagnosis of myocardial involvement. The 2024 Expert Consensus Decision Pathway recommends EMB in patients who present with SCD, VA, or high-grade AV block in patients with autoimmune disease and myocarditis [74]. Immunohistochemistry with anti-CD3, anti-CD68, and anti-human leukocyte antigen-DR (anti-HLA-DR) antibodies allow characterization of the inflammatory infiltrate in autoimmune myocarditis [74]. Combination of CMR and EMB remains important imaging modalities for early detection and management of scar- and inflammation-dependent arrhythmias in these patients.

EMB is subject to important limitations that must be acknowledged. Sampling error is inherent to the procedure, as myocardial inflammation in autoimmune disease is often focal or patchy, and a negative biopsy does not rule out myocarditis [74,75,76]. The 2024 ACC Consensus Pathway explicitly states that EMB can suffer from sampling errors, particularly when imaging demonstrates inflammation in regions that are difficult to access with standard right ventricular septal biopsy [74]. Even in clinically suspected myocarditis with cardiogenic shock, EMB yields a histologic diagnosis in only up to 74% of cases, and in recent registries, EMB was performed in fewer than 13% of adults with possible acute myocarditis [75]. Electroanatomic voltage-guided biopsy can improve sensitivity to approximately 83%, but this technique is not universally available [74]. A critical challenge specific to autoimmune myocardial disease is the spatial mismatch between EMB sampling sites and the location of abnormalities detected on CMR. Standard EMB targets the right ventricular septum near the endocardium, yet CMR studies consistently demonstrate that myocardial inflammation and fibrosis in autoimmune diseases frequently involve the subepicardial and mid-myocardial layers of the left ventricular free wall regions inaccessible to conventional biopsy [75,76]. Therefore, a negative EMB should be interpreted cautiously in the context of positive CMR findings, and the combination of CMR and EMB rather than either modality alone remains the optimal approach for early detection and management of scar- and inflammation-dependent arrhythmias in patients with autoimmune disease [74,76].

## 6. Management of Arrhythmias in Autoimmune Diseases

Arrhythmia management in autoimmune diseases includes standard antiarrhythmic therapy guided by existing guidelines and disease-specific immunosuppression targeting the inflammatory substrate that drives arrhythmogenesis (Table 1). The key principle is to control systemic inflammation which may help reduce overall arrhythmic burden [77]. Catheter ablation and device implantation (defibrillators and pacemakers) follow standard guidelines. Implantable cardiac defibrillators (ICDs) are considered an important tool among patients with SSc, especially in patients with cutaneous SSc where there is a higher risk of sudden cardiac death likely caused by malignant ventricular tachycardia [78]. However, the available literature on ICD use in patients with SSc is limited. Prior studies have found that the presence of frequent premature ventricular contractions, such as recurrent couplets, non-sustained VT, or sustained VT with or without symptoms, may represent possible risk factors for SCD [78]. Therefore, ICD implantation should be considered in patients with SSc with malignant ventricular arrhythmias either unresponsive to or with a contraindication to drug therapy [78,79]. 

Higher risk of infection, especially with devices, and recurrence risk with ablation are reported with inadequate immunosuppression [77]. In patients with Raynaud’s phenomenon, recurrent vasospasm and endothelial dysfunction create unique therapeutic considerations. Excess sympathetic activation and peripheral vasoconstriction may exacerbate digital ischemia, particularly in connective tissue diseases such as systemic sclerosis where microvascular dysfunction is central to disease pathogenesis. Consequently, antiarrhythmic therapies that promote vasoconstriction or reduce peripheral perfusion may worsen Raynaud symptoms and ischemic complications. Hydroxychloroquine, a cornerstone of SLE and RA therapy, may reduce incident AF among SLE patients (OR of 0.12, 95% CI of 0.034–0.39) [30,80]. A meta-analysis found hydroxychloroquine use is associated with QTc prolongation (OR of 1.57, 95% of CI 1.19–2.08), though large cohort studies have not demonstrated increased arrhythmia risk, and the clinical significance appears greatest in combination with other QT-prolonging medications, highlighting the importance of individualized risk assessment and ECG monitoring in higher-risk patients [81]. On the other hand, treatment of arrhythmias with selective beta blockers, sodium channel blockers or class III antiarrhythmics are best avoided due to risk of vasospasm exacerbation and digital ischemia in patients with Raynaud’s phenomenon. Optimizing therapy for Raynaud’s phenomenon prior to initiating arrhythmia therapy is crucial along with using calcium channel blockers, low-dose nonselective beta blockers, and amiodarone as alternative arrhythmia therapy.

## 7. Conclusions

**Figure 1:**
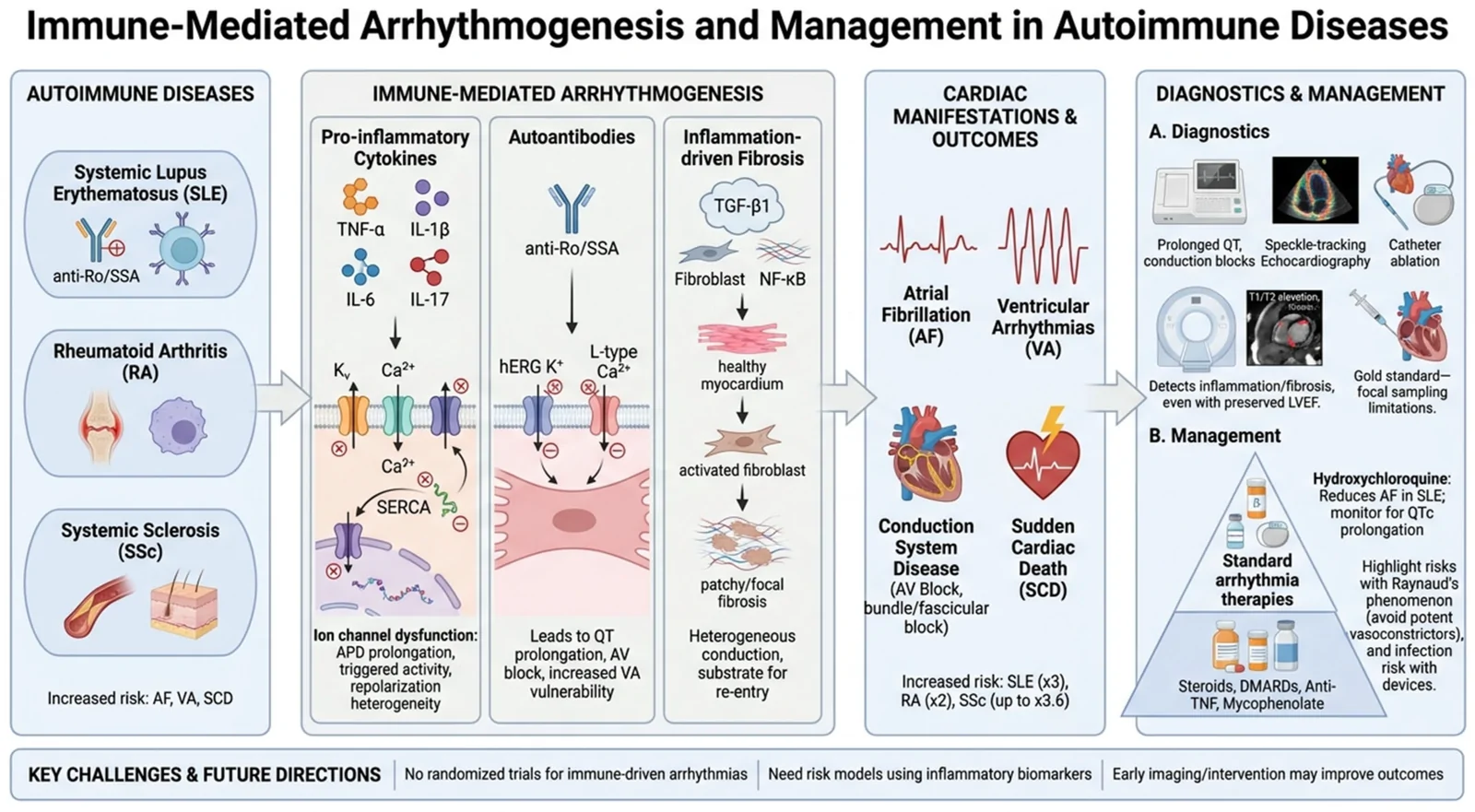
AF = atrial fibrillation, APD = action potential duration, AV = atrioventricular, Ca^2+^ = calcium ion, DMARDs = disease-modifying antirheumatic drugs, HCQ = Hydroxychloroquine, hERG K^+^ = Human Ether-à-go-go-Related Gene potassium channel, IL-1ß = Interleukin-1 Beta, IL-6 = interleukin-6, IL-17 = interleukin-17, K^+^ = potassium ion, L-Type Ca^2+^ = L-type calcium channel, LVEF = left ventricular ejection fraction, NF-KB = nuclear factor kappa-light-chain-enhancer of activated B cells, QTc = Corrected QT Interval, RA = rheumatoid arthritis, SCD = sudden cardiac death, SERCA = Sarco/Endoplasmic Reticulum Calcium ATPase, SLE = systemic lupus erythematosus, SSc = systemic sclerosis, TGF-B1 = transforming growth factor-beta 1, TNF-a = Tumor Necrosis Factor Alpha, VA = ventricular arrhythmia. All figures were conceptualized and edited by the authors and custom-created by FigureLabs (www.figurelabs.com). © FigureLabs.

Autoimmune diseases share a common arrhythmogenic substrate driven by cytokine-mediated ion channel dysfunction, autoantibody-directed conduction injury, and progressive myocardial fibrosis. Advanced CMR with parametric mapping can identify patients with active myocardial inflammation amenable to immunosuppression before irreversible fibrosis develops, and T2-weighted abnormalities improve with effective rheumatic treatment, reinforcing the importance of early intervention. Across SLE, RA, and SSc, which comprise most autoimmune diseases, immune-mediated changes lead to increased risk of AF, VA, and SCD. These are mechanistically different from ischemic etiologies and require collaborative management between rheumatologists and cardiac electrophysiologists. Randomized trials testing immunomodulatory therapies specifically for arrhythmias in autoimmune diseases are lacking. Integration of inflammatory biomarkers into arrhythmic risk prediction models, beyond LVEF alone, may allow for earlier identification of high-risk patients. Prospective registries enrolling patients across autoimmune diseases with a focus on systematic arrhythmia surveillance are essential to validate these approaches and establish evidence-based guidelines for immune-targeted antiarrhythmic therapy. In Figure 1 we have summarized the pathophysiology and cardiac manifestations autoimmune conditions can have and what diagnostics and management options we can use to move forward.

## Figures and Tables

**Table 1:** Management of arrhythmias in autoimmune diseases.

Disease	Common Arrhythmias	Immunosuppressive/Disease-Modifying Treatment	Antiarrhythmic/Device Strategy
SLE	Sinus tachycardia, AF, AV block, QTc prolongation	Severe lupus myocarditis: IV methylprednisolone → oral prednisone (1 mg/kg, tapered over max 6 months) + IV cyclophosphamide (EuroLupus protocol: 500 mg every 2 weeks × 6 doses); maintenance with mycophenolate mofetil. HCQ continued as baseline therapy. Non-severe lupus myocarditis: </strong> HCQ + oral glucocorticoids (0.5 mg/kg, tapered over max 6 months) + mycophenolate mofetil (or azathioprine).	Standard antiarrhythmics per indication. Conduction abnormalities may resolve with immunosuppression. Pacemaker for persistent high-degree AV block.
RA	AF, QTc prolongation, VA, SCD	Tight disease control with DMARDs/biologics is the primary antiarrhythmic strategy. Tocilizumab (anti-IL-6R) reduced QTc from 452 to 428 ms over 6 months, correlating with CRP reduction. Anti-TNF agents are contraindicated in pre-existing heart failure.	Beta blockers preferred (dual anti-inflammatory/antiarrhythmic effect). Standard AF management per guidelines. Caution with JAK inhibitors (CV safety signal).
SSc	PVCs (67%), NSVT (7–13%), AF/flutter (20–30%)	No SSc-specific cardiac agents; treat underlying disease with immunosuppression.	Antiarrhythmic drugs, catheter ablation, or pacemaker per standard indications.

AF: atrial fibrillation; AV: atrioventricular; HCQ: hydroxychloroquine; VA: ventricular arrhythmia; SCD: sudden cardiac death; DMARD: disease-modifying antirheumatic drugs; CRP: C-reactive protein; Anti-TNF: anti-tumor necrosis factor; JAK: Janus kinase inhibitors; CV: cardiovascular; PVCs: premature ventricular complexes; NSVT: non-sustained ventricular tachycardia.

## Data Availability

No new data were created or analyzed in this study. Data sharing is not applicable to this article.

## Abbreviations

The following abbreviations are used in this manuscript:

AF	Atrial Fibrillation
AV	Atrioventricular Block
EMB	Endomyocardial Biopsy
EP	Electrophysiology
ICD	Implantable Cardioverter Defibrillator
SLE	Systemic Lupus Erythematous
SSc	Systemic Sclerosis
SCD	Sudden Cardiac Death
VA	Ventricular Arrhythmia
